# Miscellaneous

**Published:** 1891-02

**Authors:** 


### MISCELLANEOUS.

### A QUEER CASE OF CATALEPSY.

*To the Editor of Hall's Journal of Health.*

SIR—A case has lately come to my notice which it occurred to me might be of interest to your readers. You are, doubtless, familiar with a process in dentistry known as the implantation of teeth, which is simply substitution of a natural tooth in place of a lost one, either in its vacant cavity or, as in some instances, by insertion in a new and artificially formed one.

The writer has a dentist friend who was an enthusiast regarding his profession, and especially in respect to transplantation, and like other devotees to science, he did not hesitate to experiment upon himself by inserting in his own jaw, in place of a newly extracted tooth, a veritable cat's tooth, and waited patiently upon the result of the experiment. In due time the tooth became thoroughly rooted, but the effect upon its possessor was something extraordinary. His whole demeanor underwent a change, and by no means for the better. Instead of his wonted energy and devotion to business, he showed a disposition to lounge about the house during the day time, selecting the sofa, divan, easy chairs, or even the hearth rug as a temporary dormitory, where he would lie for hours together in a kind of stupor. But no sooner would the night appear than he was all alert, manifesting great disquietude and a desire to roam abroad, particularly in the neighboring back yards, displaying great agility in scaling the fences. He would be attracted by any little scratching noise about the house, whilst the least movement of an article would cause him to dart forward, with fingers widely spread, to seize it. From being formerly a great lover of dogs, he showed an overweening dread of them, and on one occasion sought safety by shying up a telegraph pole. On moonlight evenings he was particularly ill at ease and insisted upon being admitted to the house roof, whence he would wander from roof to roof, occasionally emitting sounds in exact imitation of the fascinating invitations which romantic felines extend to one another, but now and then interjecting an angry protest as if spitefully repelling some obnoxious intruder. Finally, this state of things became unbearable, not only to the members of his immediate household, but to neighboring families in the near vicinity, and the health of my friend becoming also seriously impaired, he was persuaded to sacrifice the presumable cause of his nocturnal eccentricities. I am happy to be able to state that my friend has now partially recovered his former mental status, only now and then, under a fancied provocation, "getting his back up."

Lexington Ave., N. Y., Jan. 20. ANN AMALGUM.

NOTE.—We have admitted to our columns the foregoing communication on account of its novelty, and leave it to our readers to mentally digest and dispose it as they choose. To our mind it presents an unprecedented case of catalepsy, which we commend to the attention of investigators of psycho-animalistic phenomena.—ED.

### CLAIRVOYANCE EXTRAORDINARY.

The Rev. C. N. Barham, of Nottingham, a well known amateur of hypnotism and clairvoyance, writing to the *St. James's Gazette* of clairvoyance, among other things, says : When I resided at Whitstable, a maid servant of mine possessed this gift in a remarkable degree. At the first word of command she would fall into a deep slumber, which was accompanied by a peculiar twitching of the whole body. When in this state she could be sent—mentally, of course—from one end of England to the other.

My son was at the City of London School. Just before the vacation I desired to know how he would stand in the class list and promotion order. In order to do this I post-dated the time. The railway journey, the cab ride, and the school were accomplished. The master was interviewed; he had never seen his interlocutor. Neither does he know of the singular occult influence which environed him. The numbers were given, and given correctly.

One other extraordinary instance may be recorded. My brother-in-law was engaged to a lady in East Yorkshire. He had given her a diamond ring, which she had lost. This troubled them both. I was written to. Times and places when the ring had last been seen were given me. The girl was sent into the hypnotic sleep, and the time was ante-dated to the day when the ring had last been seen. With some trouble the sleeper was piloted through her journey to the north.

Now a new difficulty arose. I had never been to the town, did not know the house, and she was unable to find it. Conjuring up an imaginary resident, I instructed her to make the necessary inquiries. The house and the lady being found, my clairvoyant took hold of the lady's hand, watching the ring. Here and there the lady went, always accompanied by her invisible companion. At length the ring was dropped in the orchard where the engaged couple had been helping to turn over the hay. Unfortunately, the hay was being carted. In order to trace the lost ring, I commanded the girl to hold it tightly and to submit to any hardship rather than relinquish it. With a half-smile she assented, and commenced to describe her varying experiences. She told how she was raked up, handed upon a pitchfork into a hay cart, trodden upon by clowns, and eventually deposited almost at the bottom of a heap of sweet smelling hay in the corner of a disused cowhouse. Truth is stranger than fiction. Acting upon the girl's story, a search was instituted, and the ring was found. This is no romance, but a bald and disjointed record of sober facts. I could easily fill a volume with far more startling records of what may, I think, be described as extraordinary clairvoyance.—*Liverpool Courier.*

---

IN 1875, Hall's Journal of Health said : "The Packer Mfg. Co. make a soap from pure pine tar, vegetable oils and glycerine. It forms a plentiful lather, and leaves upon the skin a sense of smoothness and softness; cleanses, sweetens, deodorizes, and in dandruff, itching, eruptions, etc., it possesses healing and soothing qualities peculiar to itself. It is extensively prescribed and recommended by physicians in all parts of the country." In 1890, we are pleased to confirm our earlier commendation of Packer's Tar Soap.

### DEADLY POISONED ARROWS.

When we first encountered the tribes who fought with poisoned arrows, we were not prepared to be greatly impressed with the danger, but we received a severe lesson in August, 1887, during a fight with the Avisibba savages. Young fellows rushed with brave homicidal intentions to the front, and the tiny arrows sailed in showers past them ; but some of them found their intended billets and were arrested quivering in arms and shoulders. With contemptuous smiles the young men drew them out and flung them away, and some continued answering the savages with rifle-shots, while others sought the surgeon, bearing with them the arrows with which they had been wounded. When the day's fight was over, of course we had more leisure to examine the missiles, and our anxiety was great when we observed that they had been freshly smeared with a brown, gummy-like substance which emitted a subtle, acrid odor, with a suspicion of asafœtida in it. Quivers full of the arrows showed us that the weapons were considered by their owners to be dangerous, for those so smeared were tied together, head downward, and apart from the others.

Yet the wounds made by these slender arrows were mere punctures, such as might have been made by finely pointed butchers' skewers, and being exceedingly ignorant of the effect, we contented ourselves with syringing them with warm water and dressing them with bandages. In some instances affectionate men sucked their comrades' wounds, to make sure that nothing of the substance should be left to irritate them. In no instance was this method of any avail. All who were wounded either died after terrible sufferings from tetanus, or developed such dreadful gangrenous tumors as to incapacitate them from duty for long periods, or wreck their constitutions so completely by blood-poisoning that their lives became a burden to them.—From "The Pigmies of the Great African Forest," by HENRY M. STANLEY, in January *Scribner*.

---

AN AMERICAN GENTLEMAN, annoyed by the insignificant tallow candle furnished by the Grand Hotel, Paris, insisted upon having a *Rochester Lamp* in place of it. This was procured, and upon settling his bill, he was met with a charge of 2½ francs a night, for four months' use ; total, 320 francs ($64.00). The same lamp is sold at either the Parisian or New York 42 Park Place store of the company for $4.40.

DELICIOUS REMEDY FOR A COUGH.—Boil one ounce of flaxseed in a pint of water, strain and add a little honey, one ounce of rock candy, and the juice of three lemons; mix and boil well. Drink as hot as possible.

FOR DYSPEPSIA.—HORSFORD'S ACID PHOSPHATE.—Dr. J. J. McWilliams, Denison, Ia., says : "I have used it largely in nervousness and dyspepsia, and I consider that it stands unrivalled as a remedy in cases of this kind. I have also used it in cases of sleeplessness, with very gratifying results."

AN INTERESTING EXHIBIT in the office of A. A. Marks, the artificial limb maker, of New York, is a panoramic arrangement of envelopes addressed to him from different parts of the world, in relation to his business. They represent every modern language, and show a bewildering variety of post marks. The enterprise of this house has put them easily in the front rank of the world's makers.

### HEAD AND HAIR OF INFANTS.

The heads of infants should not be washed in brandy, whisky, spirits of hartshorn, or other stimulating washes. They do no good, cause pain, and may so irritate the tender scalp as to cause disease.

For cleansing the head, soap and water, or water with a little borax in it are all that is needed. After washing the scalp a soft hair brush should be used. This will remove any dirt or dandruff, and will not irritate the skin as a comb would be likely to do.

The hair of both boys and girls should be kept short till eight or nine years of age. This will conduce to cleanliness; prevent a great deal of trouble in combing and washing; will leave no harbor for the abominable creepers to which children are exposed; and by keeping the head cool will render children less liable to the inflammatory affections of the brain—to which they are strongly predisposed at their time of life. Thus managed, the hair will be smooth and glossy, and accumulations on the scalp will be prevented.

The custom of putting caps on infants having been abolished by all well informed people, it is hardly necessary to say that the practice should be abandoned by all, as the head is warm enough without the cap, is very likely to be too warm with it, and in this way causing the brain affections to which children are so prone.

---

HICCOUGH.—For this affection, which is so often provokingly obstinate, close the ears with the tips of the fingers, exert a certain pressure therewith, and at the same time, drink, in small draughts, any sort of a liquid offered by another person and the hiccough will cease instantaneously.

BOWEL DIFFICULTY IN CHILDREN.—The following simple suggestions will avoid nearly, if not all, bowel difficulties in children, not only in summer, but at all other times of the year. First, make sure of pure water, and if you are at all doubtful about the purity of your supply, boil it well before using. Boil the milk thoroughly, and have all food properly sterilized by cooking. Avoid the use of green fruit. Ripe, fresh fruit may be eaten freely at meals, but at no other time; yet there are thousands of persons who never think of eating fruit except between meals. This imposes extra burdens upon the stomach, and makes many dyspeptics. The stomach gets tired out by being continually worked, and by and by declares a "strike" in the shape of sick headache. The constant use of any muscle will induce excessive fatigue. Try moving the hand gently but constantly for twenty-four consecutive hours, and you will gain a clearer meaning of what a task you impose upon your stomach by keeping it constantly at work. It is a muscular organ and should receive the consideration given to muscles less delicate.

ORTHODOXY is the Bourbon of the world of thought. It learns not, neither can it forget; and though, at present, bewildered and afraid to move, it is as willing as ever to insist that the first chapter of Genesis contains the beginning and end of sound science, and to visit with such petty thunderbolts as its half paralyzed hands can hurl at those who refuse to degrade nature to the level of primitive Judaism.—*Professor Huxley.*

### A BIO-MAGNETIC INSTITUTE IN NEW YORK CITY.

Dr. Josef Grigorowitsch, from Moscow, Russia, is a Magneto-Therapeutist of great repute in his own country as well as in Germany, where he has demonstrated his capacity to heal disease by his therapeutic methods as attested by recomendations from people of the highest social position. Having resided for some time in India, Dr. Grigorowitsch became familiar with the psycho-magnetic process of healing as practised by those wonderful native mystic-therapeutists, and has adopted their modus operandi in his practice which differs from the usual methods of the show-magnetiser or hypnotiser. Dr. Grigorowitsch's method seems to produce a more gentle equalization of the physiological working in the human organism, tending to secure a permanent re-establishment of a normal and healthful state in the organism of his patients. Dr. Grigorowitsch has a free Clinic for the poor every Saturday, from 3 to 5 P. M., at his residence, 683 Lexington avenue, N. Y., and hopes to open an institution for the psycho-magnetic-therapics if encouraged by people of means interested in this method of healing, where instruction in the various departments of psycho-magnetic-therapic will be given.

---

### TOPOLOBAMPO.

We have hitherto admitted articles pro and con the Topolobampo scheme to our columns, but so far from encouraging it, the JOURNAL always took decided ground against its practicability. It seemed ill-conceived and highly visionary at the best. An item which recently appeared in the Chicago R. P. Journal respecting this particular enterprise, abundantly fortifies our position.

The United States as a country is good enough for the most of us.

---

DR. T. B. WESTWOOD, *of Brooklyn, N. Y.*, writes : "I consider *Bromo-Seltzer* one of the *best* preparations on the market for the relief of those distressing *headaches* arising from nervous troubles, and especially in those forms to be met with in *hysterical* and *over-worked* women." We call your special attention to Bromo-Seltzer card on page vii.

WEAK WRIST.—In young, immature people, rapidly completing their growth, over-exertion is often attended with consequences more serious and lasting than at first expected. In this case, dumb-bells obviously of a weight beyond the strength or capacity of the muscles were used, with the result—a not uncommon one—that inflammation of the sheath of the extensor tendons was set up, with exudation of fluid, and the presence in the sheath of that fluid gives rise to great inconvenience. The first thing is to disperse the ganglion, and therein lies the difficulty. A shilling can be put upon it, and a smart blow can be given it, or a book can be placed violently upon the ganglion ; sometimes that will disperse the fluid, and in a month, with the help of frequent bathing and gentle friction, the wrist becomes quite strong. But if the violent blow is insufficient, as it often is, then a coin should be strapped firmly upon the ganglion and kept there for a week, or iodine can be painted over the spot. Usually, however, self treatment is not sufficient, as thousands of young servants similarly affected find out every year.

